# Acute social jetlag augments morning blood pressure surge: a randomized crossover trial

**DOI:** 10.1038/s41440-023-01360-5

**Published:** 2023-07-14

**Authors:** Nobuhiro Nakamura, Hiroshi Akiyama, Mei Nishimura, Kejing Zhu, Katsuhiko Suzuki, Mitsuru Higuchi, Kumpei Tanisawa

**Affiliations:** 1 Faculty of Sport Sciences, Waseda University, Tokorozawa, Saitama Japan; 2 Graduate School of Sport Sciences, Waseda University, Tokorozawa, Saitama Japan; 3 School of Sport Sciences, Waseda University, Tokorozawa, Saitama Japan

**Keywords:** Central arterial stiffness, Morning blood pressure surge, Social jetlag

## Abstract

Although social jetlag (SJL) is generally considered a chronic condition, even acute SJL may have unfavorable effects on the cardiovascular system. We focused on the acute effects of SJL on morning blood pressure (BP) surge. This randomized crossover trial recruited 20 healthy men. In the SJL trial, participants delayed their bedtime by three hours on Friday and Saturday nights. Participants in the control (CON) trial implemented the same sleep-wake timing as on weekdays. Pre- and post-intervention measurements were performed to evaluate resting cardiovascular variables on Friday and Monday mornings, respectively. The ambulatory BP was automatically measured during the sleep and awake periods for 2 h after the participant woke up at night before pre- and post-intervention measurements. SJL (average mid-sleep time on weekends – average mid-sleep time on weekdays) occurred only in the SJL trial (SJL: 181 ± 24 min vs. CON: 8 ± 47 min). Carotid-femoral pulse wave velocity (cfPWV) and morning BP surge on Monday in the SJL trial were significantly higher than those on Friday in the SJL trial (cfPWV: *P* = 0.001, morning BP surge: *P* < 0.001), and those on Monday in the CON trial (cfPWV: *P* = 0.007; morning BP surge: *P* < 0.001). Furthermore, a significant positive correlation was found between ΔcfPWV and Δmorning BP surge (*R* = 0.587, *P* = 0.004). These results suggest that even acute SJL augments morning BP surge. This phenomenon may correspond to increased central arterial stiffness.

**State the details of Clinical Trials:** Name: Effect of acute social jetlag on risk factors of lifestyle-related diseases. URL: https://center6.umin.ac.jp/cgi-open-bin/ctr_e/ctr_view.cgi?recptno=R000053204. Unique identifier: UMIN000046639. Registration date: 17/01/2022

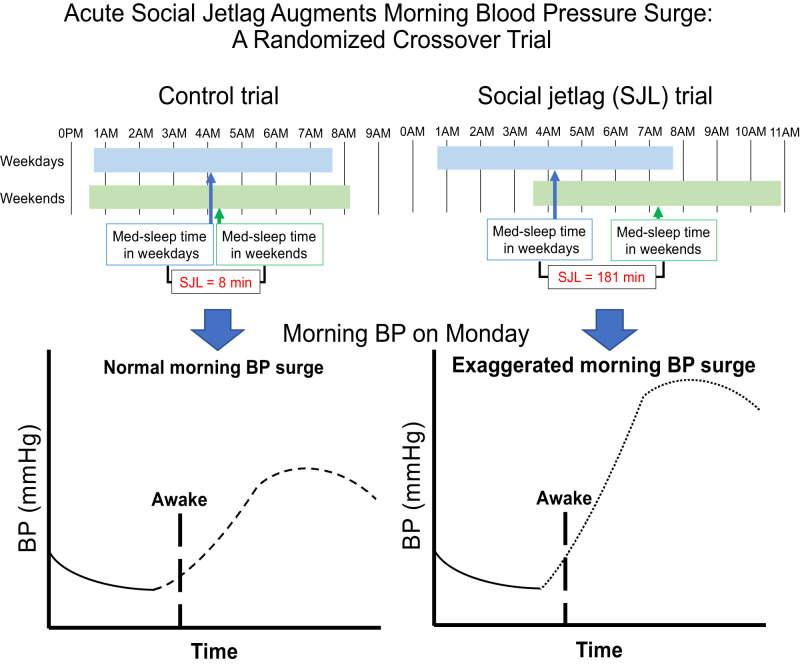

## Introduction

The discrepancy in sleep–wake timing between workdays and free (non-work) days due to modern work schedules possibly causes mild circadian misalignment between endogenous circadian rhythm and the sleep schedule imposed by social obligations. This jetlag-like phenomenon is called “social jetlag (SJL),” [1] which is measured as the differences between mid-sleep time on workdays and free days [1, 2]. SJL is prevalent in the general population [3–5] and has been suggested to lead to cardiovascular diseases [6, 7]. Although SJL is generally considered a chronic condition [3–5], even acute SJL may have unfavorable effects on the cardiovascular system, given that the simulated jetlag due to air travel acutely affects the cardiovascular system in an animal study [8]. To the best of our knowledge, no evidence shows that acute SJL influences the cardiovascular system in humans.

In humans, blood pressure (BP) shows circadian variations; it decreases during sleep and increases in the morning [9, 10]. The diurnal variation in pressure stress from the nadir during sleep to the peak in the morning is called the morning BP surge. A normal morning BP surge is a physiological phenomenon, whereas an exaggerated morning BP surge is an independent predictor of cardiovascular events [11, 12]. Interestingly, the morning BP surge has a weekly variation and is most significant on Monday [13]. In modern society, many people work on weekdays (Monday to Friday) and take a day off on weekends (Saturday and Sunday); thus, the weekly variation may be influenced by acute SJL on weekends.

Thus, the purpose of the present study was to investigate the acute effects of SJL on morning BP surge. As an unhealthy lifestyle on free days, such as excess intake of energy as well as reduced physical activity levels, may confound the effects of SJL, we designed a randomized crossover trial to eliminate these potential confounding effects. We hypothesized that even acute SJL augments morning BP surge. Furthermore, the present study evaluated the effects of acute SJL on other cardiovascular variables and the autonomic nervous system, such as central arterial stiffness and sympathetic nerve activity in a vessel, to elucidate the underlying physiological mechanisms of the morning BP surge due to acute SJL.

## Materials and methods

### Participants

Twenty healthy men were recruited to participate in this study. All participants did not have a history of hypertension (systolic BP [SBP] ≤ 140 and diastolic BP [DBP] ≤ 90 mmHg), were nonsmokers, and were free of overt cardiovascular disease or diabetes, as assessed by medical history. All participants provided written informed consent to participate before the start of this study. The purpose, procedures, and risks involved in this study were reviewed and approved by the Human Research Committee of Waseda University (approval No. 2021-214). This study was conducted according to the guidelines of the 1975 Declaration of Helsinki.

### Experimental design

The present study was an open-label, randomized crossover trial consisting of the SJL and control (CON) trials (Figure S1). The study protocol is registered in the University Hospital Medical Information Network-Clinical Trial Registry (number: UMIN000046639, registration date: 17/01/2022). The present study shows a part of the primary outcomes listed in the registered protocol. Figure S2 shows the schedule of the present study. The participants were randomly assigned to the CON or SJL trials after the screening term. One investigator was responsible for the randomization and allocation of the participants to two trials. The allocation list was created using spreadsheet software (Microsoft Excel, Microsoft, WA, USA).

After written informed consent was obtained, wearable devices (Fitbit Charge 5, Fitbit, CA, USA) were distributed to the participants to monitor sleep variables and energy balance throughout this study. The device automatically measures bedtime, awake time, sleep duration (percentage of time in rapid eye movement [REM], light and deep sleep), and daily total energy expenditure. The validity of Fitbit Charge series for measuring the sleep variables has been confirmed by comparison with polysomnography and actigraphy [14, 15]. The energy expenditure for every 5 min was extracted, and the amount of physical activity for 2 h before and after awake time was calculated from the energy expenditure. The participants were instructed about the correct usage of the device (Fitbit: Health & Fitness, Fitbit, CA, USA) and to record food intake by themselves. The participants were allotted a period to familiarize themselves with the device and the application. We required the participants to wear the device during the intervention period, except for bathing. The intervention was conducted for 4 weeks, as described below.

#### Week one

We started with a screening term to confirm sleep variables (bedtime, awake time, and mid-sleep time [median time between bedtime and awake time]) under free-living conditions for each participant from Wednesday in week one to Tuesday in week two. We confirmed that day-to-day intraindividual variation in mid-sleep time on weekdays was within 2 h.

#### Week two

Resting hemodynamics, autonomic nervous system, cardiovagal baroreflex sensitivity (cvBRS), and central arterial stiffness were measured on Friday morning as pre-intervention measurement one. The participants were required to perform the SJL or CON trial from Friday night to Sunday morning.

#### Week three

Post-intervention measurement one was conducted on Monday morning, as with pre-intervention measurement one. Afterward, the participants were required to live according to everyday routine until Thursday as a washout term. Pre-intervention measurement two was conducted on Friday morning. The participants were then crossed over to the opposite trial and were instructed to have the same meal and to perform physical activity as per the last weekend to avoid the confounding effect of energy expenditure and intake.

#### Week four

Post-intervention measurement two was performed on Monday morning. After that, the participants were required to live according to routine life until Thursday as a follow-up.

#### Social jetlag and control trials

In the SJL trial, the participants were required to delay their bedtime by 3 h on Friday and Saturday nights in week two or three (Figure S3). The participants in the CON trial were instructed to implement the same sleep–wake timing as on weekdays. These sleep–wake timings during the intervention were based on those during the screening term. SJL was calculated as follows: SJL (min) = average mid-sleep time on weekends in week two or three – average mid-sleep time on weekdays in the screening term.

#### Pre- and post-intervention measurements

Pre- and post-intervention measurements were performed on Friday and Monday mornings; the participants were tested at the same time of day throughout the study period to avoid potential diurnal variations. Before these measurements, the participants were instructed to avoid food and caffeine and alcohol intake for at least 12 and 24 h, respectively. Pre- and post-intervention measurements were conducted under comfortable laboratory conditions between 09:00 AM and 11:00 AM.

### Body composition

The percentage of body fat and lean body mass was measured using bioelectrical impedance analysis (InBody 720, InBody, Tokyo, Japan) with the participants in the upright position. The validity of the device for measuring body composition has been confirmed by comparison with dual-energy x-ray absorptiometry, which is the gold standard for measuring body composition [16–18].

### Central arterial stiffness

The participants were studied under quiet resting conditions for 10 min in the supine position. Carotid-femoral pulse wave velocity (cfPWV), which is the gold standard for measuring central arterial stiffness [19, 20], was measured using a vascular testing device (form PWV/ABI, Omron Colin, Tokyo, Japan). Carotid arterial pressure waveforms were stored for 30 s using applanation tonometry sensors attached to the right common carotid and common right femoral arteries. The value of cfPWV was calculated using the length from the heart to the common carotid (Lhc) and common femoral arteries (Lhf) and the transit time (Tcf): [21]

$${{{{{\rm{cfPWV}}}}}}=\frac{{Lhf}-{Lhc}}{{{{{{\rm{Tcf}}}}}}}$$

The coefficient of variation for the cfPWV was 4.9 ± 4.1% within the same participant.

### Ambulatory blood pressure and heart rate

The ambulatory BP monitoring (ABPM) device (TM-2441, A&D Medical, Tokyo, Japan) automatically measured ambulatory BP and heart rate (HR) using the oscillometric method every 30–60 min during the sleep and awake period until measurement was completed for 2 h after wake-up. The ambulatory BP monitoring device has been confirmed to be accurate and fulfilled all ISO 81060-2:2013 standard requirements for ABPM determination in adults [22]. The measurement was automatically performed the night before pre-intervention measurement (Thursday night to Friday morning in weeks two and three) and post-intervention measurement (Sunday night in weeks two and three to Monday morning in weeks three and four).

Sleep BP was defined as the average BP readings during sleep. The lowest BP was defined as the average of three BP readings (1.5 h) during sleep. Preawakening BP was defined as the average BP readings during the 2 h just before awake time (four BP readings). Morning BP was defined as the average BP readings during the first 2 h after the awake time (four BP readings) (Figure S4) [11]. Then, the morning BP surge was calculated as follows: [11]

$${{{{{\rm{Morning}}}}}}\; {{{{{\rm{BP}}}}}}\; {{{{{\rm{surge}}}}}}={{{{{\rm{morning}}}}}}\; {{{{{\rm{systolic}}}}}}\; {{{{{\rm{BP}}}}}}({{{{{\rm{SBP}}}}}})-{{{{{\rm{lowest}}}}}}\; {{{{{\rm{nocturnal}}}}}}\; {{{{{\rm{SBP}}}}}}$$

$${{{{{\rm{Preawakening}}}}}}\; {{{{{\rm{morning}}}}}}\; {{{{{\rm{BP}}}}}}\; {{{{{\rm{surge}}}}}}={{{{{\rm{preawakening}}}}}}\; {{{{{\rm{SBP}}}}}}-{{{{{\rm{lowest}}}}}}\; {{{{{\rm{nocturnal}}}}}}\; {{{{{\rm{SBP}}}}}}$$

$${{{{{\rm{Wakening}}}}}}\; {{{{{\rm{morning}}}}}}\; {{{{{\rm{BP}}}}}}\; {{{{{\rm{surge}}}}}}={{{{{\rm{morning}}}}}}\; {{{{{\rm{SBP}}}}}}-{{{{{\rm{preawakening}}}}}}\; {{{{{\rm{SBP}}}}}}$$

The coefficients of variation for the morning SBP and the lowest nocturnal SBP were 7.1 ± 6.6% and 7.0 ± 7.3% within the same participant, respectively [23].

### Cardiovagal baroreflex sensitivity

Spontaneous cardiovagal baroreflex sensitivity (cvBRS) was determined from beat-to-beat changes in R–R interval (RRI) and SBP using an analysis software (Beatscope, version 1.1, Finapres Medical Systems, Amsterdam, The Netherlands). Three or more beats of progressive SBP changes and corresponding changes of RRI were identified as baroreflex sequences. Both were recorded in the present study. The minimum criteria for accepting a sequence were set at 1 mmHg for SBP and 4 ms for RRI. The slope of the linear correlation between RRI and SBP was assessed for up–up (progressive increases of SBP followed by a lengthening of the RRI) and down–down (progressive decreases of SBP with a subsequent shortening of the RRI) cvBRSs, which was determined if the r-value was > 0.8. Both cvBRSs were averaged for each participant for the last 5 min of 10-min rest [24].

### HR and BP variabilities

HR variability (HRV) was measured as a reliable quantitative marker to assess the activity of the sympathetic and parasympathetic branches of the autonomic nervous system using HRV analysis module (MLS 310, AD Instruments, New South Wales, Australia). In the time domain, the root mean square of the squared differences of successive RRI (RMSSD) estimates short-term variation of HR caused by parasympathetic activity [25]. In the frequency domain analysis, the variance of the signal is calculated through a short, fast Fourier transform, and, according to the frequency band classification, it is characterized into low-frequency (LF: 0.04–0.15 Hz) and high-frequency (HF: 0.15–0.4 Hz). The power component of the LF band includes sympathetic and parasympathetic influences. HF band is primarily influenced by the efferent activity of the vagal tone [25]. The ratio between normalized LF and HF (LF/HF) is established as an index of the sympathovagal balance [26].

BP variability was assessed as a marker of efferent sympathetic vascular modulation [27]. A previous study has reported that oscillation in the LF component of the SBP power spectral density (LF-SBP) is associated with an increase in the sympathetic drive [28]. The LF-SBP was evaluated in the frequency domain using an analysis module (MLS 370, AD Instruments, New South Wales, Australia), similar with HRV analysis.

### Hemodynamics in rest

HR and beat-to-beat BP waveform were monitored using a three-lead electrocardiogram (BSM-2401, Nihon Kohden, Tokyo, Japan) and finger photoplethysmography (Finometer MIDI, Finapres Medical Systems, Amsterdam, The Netherlands), respectively. The latter instrument was attached to the middle finger of the left hand. The BP value obtained from the finger was automatically reconstructed as the brachial BP value (Beatscope, version 1.1, Finapres Medical Systems, Amsterdam, The Netherlands) [29]. The method has been validated by comparison with intra-arterial brachial BP [29]. Furthermore, stroke volume (SV) was calculated based on the obtained BP waveform using the model flow method [30], which incorporates age, height, and body mass and simulates aortic flow waveforms from an arterial pressure signal using a nonlinear three-element model of the aortic input impedance (Beatscope, version 1.1, Finapres Medical Systems, Amsterdam, The Netherlands). The method has been validated by comparison with the thermodilution method [30]. Cardiac output (CO) and total peripheral resistance (TPR) were then calculated as SV × HR and mean arterial pressure (MAP)/CO, respectively. In addition, a carotid pressure waveform was obtained in the right common carotid artery. The pressure waveforms were converted from an oscillometric device (form PWV/ABI, Omron Colin, Komaki, Japan) at a sampling rate of 1000 Hz through an analog/digital converter (PowerLab/16SP, AD Instruments, New South Wales, Australia) and recorded in a device connected to a personal computer (Macbook Pro, Apple, CA, USA). Then, the obtained data were analyzed using an analysis software (LabChart8, AD Instruments, New South Wales, Australia). The carotid arterial pressure was calibrated by equating the carotid DBP and MAP to the brachial artery value [31]. Then, these hemodynamics variables were measured for 60 s after resting in the supine position for 10 min. The coefficient of variation for the carotid SBP was 6.1 ± 4.1% within the same participant.

### Statistical analyses

A priori statistical power analysis was performed to determine the sample size needed for the present study using G*Power 3.1.9.6 [32]. We determined that a sample size of 12 was needed to achieve a statistical power (1 − *β*) of more than 0.80, which was required to reject the null hypothesis, with a large standardized effect size (*f* = 0.40) and an error probability of 0.05 (*α*), using a repeated measure two-way analysis of variance (ANOVA) (number of trials = 2, number of measurements = 2). Although the minimum calculated sample size was 12, we recruited 20 participants considering the possibility of discontinued intervention or missing data to measurement errors.

All data are expressed as mean ± standard deviation (SD). Statistical analyses were performed using IBM SPSS Statistics for Mac version 27.0 (IBM Corp., Armonk, N.Y., USA). The effects of time and trials were examined by the two-way repeated-measures ANOVA (trial × time) for participants’ characteristics, ambulatory BP and HR, hemodynamics, baroreflex sensitivity, autonomic nervous system, central arterial stiffness, and morning BP surge. The difference in the time course of ambulatory SBP and physical activity between the trials was also analyzed by the two-way repeated-measures ANOVA. In the case of significant *F* values, it was analyzed using Bonferroni’s post hoc test. One-way repeated-measures ANOVA was applied to compare the differences in sleep variables, including SJL and energy balance, between weekdays (during the screening term) and weekends (during the screening term, the SJL and CON trials).

Moreover, we calculated the absolute change (Δ) from Friday to Monday in each trial in cfPWV, morning BP surge, and LF-SBP. Then, Pearson’s correlation coefficients were used to assess the relationships between the ΔcfPWV and Δmorning BP surge or ΔLF-SBP, Δmorning BP surge, and ΔLF-SBP. The level of significance for all comparisons was set at *P* < 0.05.

## Results

### Data exclusion

Six participants were excluded from this study because four showed SJL of < 2 h in the SJL trial, and the ambulatory BP monitoring device did not work for two participants. Therefore, results were based on the remaining 14 participants who completed all measurements (Figure S1). To assess whether the results were affected by excluding participants, we also analyzed the morning BP surge, cfPWV, and the time course of ambulatory SBP using the data from all participants, including those who did not adhere to the intervention in the SJL trial.

### Participants’ characteristics

Table 1 shows the participants’ characteristics of the present study. The two-way ANOVA revealed no significant trial × time interactions for all participant characteristics (body mass *F* = 3.931, *P* = 0.069; body mass index [BMI]: *F* = 4.452, *P* = 0.055; body fat: *F* = 0.324, *P* = 0.579; lean body mass: *F* = 0.001, *P* = 0.974; waist circumference: *F* = 0.784, *P* = 0.392; intracellular water: *F* = 0.051, *P* = 0.825; extracellular water: *F* = 0.009, *P* = 0.927).

**Table 1:** Participants’ characteristics

	CON		SJL		*P* value		
	Friday	Monday	Friday	Monday	Interaction	Trial	Time
Age, y	26 (3)				n/a		
Height, cm	174.2 (6.2)						
Body mass, kg	69.8 (10.6)	69.9 (10.7)	69.9 (10.7)	69.7 (10.5)	0.069	0.690	0.871
BMI, kg/m^2^	23.0 (3.1)	23.0 (3.2)	23.0 (3.2)	22.9 (3.1)	0.055	0.366	0.703
Body fat, %	16.8 (5.9)	17.0 (5.5)	17.7 (7.9)	17.4 (5.7)	0.579	0.109	0.791
Lean body mass, kg	57.7 (7.1)	57.7 (7.1)	57.0 (7.4)	57.2 (7.0)	0.974	0.066	0.973
Waist circumference, cm	78.1 (8.1)	77.8 (8.4)	78.0 (8.1)	77.9 (8.3)	0.392	0.969	0.211
Intracellular water, L	26.3 (3.2)	26.3 (3.2)	26.0 (3.4)	26.1 (3.2)	0.825	0.144	0.674
Extracellular water, L	15.6 (2.1)	15.6 (2.0)	15.5 (2.1)	15.5 (2.0)	0.927	0.151	0.944

Values are presented as mean (standard deviation)

*BMI* body mass index, *CON* control trial, *SJL* social jetlag trial

### Sleep variables and energy balance

Sleep variables and energy balance during the screening term, the SJL trial, and the CON trial are summarized in Table 2. One-way ANOVA indicated a significant difference in bedtime, awake time, and mid-sleep time on the weekend in the SJL trial compared with weekdays in the screening term, weekend in the screening term, and CON trial (bedtime: *P* = 0.014; awake time: *P* < 0.001; mid-sleep time: *P* < 0.001). SJL was significantly different in the SJL trial compared with the CON trial (SJL: 181 ± 24 min vs. CON: 8 ± 47 min or weekend in screening term: 27 ± 60 min). On the other hand, there were no significant differences in bedtime, sleep duration, REM sleep, light sleep, deep sleep, energy expenditure, and intake on weekends in the SJL trial compared with weekdays in the screening term (sleep duration: *P* = 0.588; REM sleep: *P* = 0.827; light sleep: *P* = 0.945; deep sleep: *P* = 0.879; energy expenditure: *P* = 0.600; energy intake: *P* = 0.964). No significant differences in sleep variables and energy compared with weekdays in the screening term were observed on the weekend of the CON trial.

**Table 2:** Sleep variables and energy balance

	Weekday in screening term	Weekend in screening term	Weekend in CON	Weekend in SJL	*P* value
Bedtime, h:m	0:43 (0:56)	0:58 (0:58)	0:36 (1:06)	3:39 (1:03)^a,b,c^	0.014
Awake time, h:m	7:48 (0:35)	8:27 (0:52)	8:11 (1:06)	10:53 (0:49)^a,b,c^	< 0.001
Med-sleep time, h:m	4:15 (0:39)	4:41 (0:44)	4:23 (0:58)	7:16 (0:50)^a,b,c^	< 0.001
Social jetlag, min		27 (60)	8 (47)	181 (24)^b,c^	< 0.001
Sleep duration, min	368 (49)	373 (60)	390 (53)	377 (58)	0.588
REM sleep (%)	20.8 (4.1)	20.6 (8.1)	21.2 (7.1)	21.8 (6.6)	0.827
Light sleep (%)	60.7 (5.1)	60.6 (7.9)	60.3 (7.6)	59.2 (8.1)	0.945
Deep sleep (%)	18.5 (3.5)	18.8 (4.2)	18.5 (3.3)	19.0 (4.8)	0.879
Energy expenditure, kcal	2897 (462)	2823 (528)	2885 (528)	2795 (457)	0.600
Energy intake, kcal	2025 (455)	2049 (686)	2049 (499)	2075 (550)	0.964

Values are presented as mean (standard deviation)

*REM* rapid eye movement

^a^Significantly different from weekday in screening term (*P* < 0.05)

^b^Significantly different from weekday in screening term (*P* < 0.05)

^c^Significantly different from the weekend in the control trial (*P* < 0.05)

### Ambulatory blood pressure, heart rate, and physical activity

Table S1 indicates ambulatory BP and HR on Friday and Monday in both trials. There was a significant trial × time interaction in morning SBP and double product (morning SBP: *F* = 20.066, *P* = 0.001; morning DBP: *F* = 5.019, *P* = 0.043; preawakening DBP: *F* = 6.809, *P* = 0.022; preawakening HR: *F* = 19.343, *P* = 0.001; morning double product: *F* = 4.769, *P* = 0.048; wakening morning BP surge: *F* = 5.393, *P* = 0.037). Both variables on Monday in the SJL trial were significantly higher than those on Friday in the SJL trial (morning SBP: *P* < 0.001; morning double product: *P* = 0.008) and those on Monday in the CON trial (morning SBP: *P* < 0.001; morning double product: *P* = 0.026). In contrast, other ambulatory hemodynamics showed no significant interaction (asleep SBP: *F* = 1.784, *P* = 0.204; asleep DBP: *F* = 0.156, *P* = 0.700; asleep HR: *F* = 0.618, *P* = 0.446; preawakening SBP: *F* = 3.258, *P* = 0.094; morning HR: *F* = 0.004, *P* = 0.950; the lowest nocturnal SBP: *F* = 0.001, *P* = 0.985; preawakening morning BP surge: *F* = 0.443, *P* = 0.517).

**Fig. 1:**
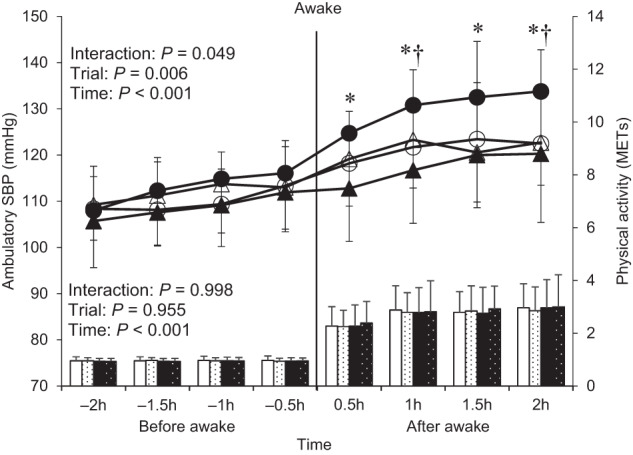
Time course of ambulatory systolic blood pressure (line chart) and physical activity (vertical bar chart) for 2 h before and after the awake time (n = 14). A vertical bar line means the awakening point as zero. Line charts show the time course of ambulatory systolic blood pressure as follows: open triangle (△): CON trial on Friday, open circle (○): CON trial on Monday, closed triangle (▲): SJL trial on Friday, closed circle (●): SJL trial on Monday. Vertical bar charts show as follows: white bar: CON trial on Friday, white bar with a black dot: CON trial on Monday, black bar: SJL trial on Friday, black bar with a white dot: SJL trial on Monday. CON, control trial; SBP, systolic blood pressure; SJL, social jetlag trial. Values are presented as mean ± standard deviation. ^*^Significantly different from Friday in the SJL trial at the same point (*P* < 0.05). ^†^Significantly different from Monday in the CON trial at the same point (*P* < 0.05)

Figure 1 summarizes the difference in the time course of ambulatory SBP (line chart) and physical activity (vertical bar chart) for 2 h before and after an awake time between the trials. We also showed individual data of the time course of ambulatory SBP during CON and SJL trials from Sunday night to Monday morning in Figure S5. Significant trial × time interaction was observed for ambulatory SBP (*F* = 1.601, *P* = 0.049). There was no significant trial × time interactions in physical activity (*F* = 0.072, *P* = 0.998). Ambulatory SBP on Monday in the SJL trial was significantly higher than that on Friday in the SJL trial at all points after wake-up (0.5 h: *P* = 0.007; 1 h: *P* = 0.006; 1.5 h: *P* = 0.018; 2 h: *P* = 0.019) and Monday in the CON trial at 1 h and 2 h after wake-up (1 h: *P* = 0.035; 2 h: *P* = 0.046). In contrast, the two-way ANOVA revealed no significant trial × time interactions in the time course of ambulatory SBP (*F* = 1.365, *P* = 0.132, n = 18) when including those who did not adhere to the intervention in the SJL trial (< 2 h).

### Hemodynamics, baroreflex sensitivity and autonomic nervous system

Table 3 shows the hemodynamics, baroreflex sensitivity, and autonomic nervous system response to SJL. Significant trial × time interaction was observed for brachial and carotid SBP and TPR (brachial SBP: *F* = 5.691, *P* = 0.033; carotid SBP: *F* = 4.946, *P* = 0.044; TPR: *F* = 5.232, *P* = 0.040). LF-SBP showed significant trial × time interaction (*F* = 8.023, *P* = 0.014). Post hoc test revealed that the brachial SBP and LF-SBP on Monday in the SJL trial were significantly higher than those on Friday in the SJL trial (brachial SBP: *P* = 0.001; LF-SBP: *P* = 0.015) and those on Monday in the CON trial (brachial SBP: *P* = 0.003; LF-SBP: *P* = 0.018). Carotid SBP on Monday in the SJL trial was significantly higher than on Friday in the SJL trial (*P* = 0.002). There were no significant trial × time interactions with other hemodynamics, baroreflex sensitivity, and autonomic nervous system (brachial DBP: *F* = 0.641, *P* = 0.438; MAP: *F* = 4.092, *P* = 0.064; carotid PP: *F* = 3.955, *P* = 0.068; HR: *F* = 0.015, *P* = 0.905; SV: *F* = 1.145, *P* = 0.304; CO: *F* = 1.187, *P* = 0.296; double product: *F* = 1.773, *P* = 0.206; RMSSD: *F* = 0.078, *P* = 0.785; LF/HF: *F* = 1.304, *P* = 0.274; cvBRS up–up: *F* = 0.013, *P* = 0.912; cvBRS down–down: *F* = 0.225, *P* = 0.643).

**Table 3:** Hemodynamics and autonomic nervous system at rest

	CON		SJL		*P* value		
	Friday	Monday	Friday	Monday	Interaction	Trial	Time
Brachial SBP, mmHg	108 (6)	110 (5)	108 (8)	118 (9)^a,b^	0.033	0.002	0.034
Brachial DBP, mmHg	61 (7)	61 (6)	59 (8)	60 (4)	0.438	0.297	0.781
MAP, mmHg	80 (7)	80 (6)	78 (8)	82 (8)	0.064	0.757	0.124
Brachial PP, mmHg	47 (7)	50 (6)	49 (6)	58 (8)	0.056	0.120	0.006
Carotid SBP, mmHg	105 (6)	106 (6)	104 (7)	111 (11)^a^	0.044	0.427	< 0.001
Carotid PP, mmHg	44 (8)	45 (7)	45 (6)	51 (9)	0.068	0.145	0.047
HR, bpm	59 (9)	59 (7)	58 (9)	59 (11)	0.905	0.952	0.834
SV, mL	104 (16)	107 (18)	104 (15)	102 (25)	0.304	0.337	0.974
CO, L/min	6.1 (1.4)	6.3 (1.5)	6.1 (1.4)	5.9 (1.5)	0.296	0.405	0.964
TPR, mmHg/L/min	13.7 (3.1)	13.3 (2.9)	13.4 (3.2)	14.8 (3.9)	0.040	0.276	0.328
Double product, mmHg×bpm	6302 (916)	6463 (762)	6278 (911)	6935 (1565)	0.206	0.622	0.120
RMSSD, ms	74.9 (45.4)	71.8 (52.7)	71.8 (43.0)	72.9 (40.0)	0.785	0.891	0.876
LF/HF	1.2 (1.1)	1.3 (1.1)	1.3 (1.1)	1.1 (0.8)	0.274	0.759	0.823
LF-SBP, mmHg^2^/Hz	1.5 (0.9)	1.3 (1.2)	1.4 (1.1)	2.8 (2.2)^a,b^	0.014	0.076	0.037
cvBRS up-up, ms/mmHg	20.7 (12.7)	21.6 (9.5)	21.3 (11.8)	21.6 (9.4)	0.912	0.922	0.797
cvBRS down-down, ms/mmHg	21.4 (9.9)	21.8 (9.4)	20.7 (10.6)	22.1 (10.0)	0.643	0.960	0.468

Values are presented as mean (standard deviation)

*CO* cardiac output, *CON* control trial, *cvBRS* cardiovagal baroreflex sensitivity, *DBP* diastolic blood pressure, *HF* high frequency, *HR* heart rate, *LF* low frequency, *LF-SBP* low frequency-systolic blood pressure, *MAP* mean arterial pressure, *PP* pulse pressure, *SBP* systolic blood pressure, *SJL* social jetlag trial, *SV* stroke volume, *TPR* total peripheral resistance

^a^Significantly different from Friday in the SJL trial (*P* < 0.05)

^b^Significantly different from Monday in the CON trial (*P* < 0.05)

### Central arterial stiffness and morning BP

**Fig. 2:**
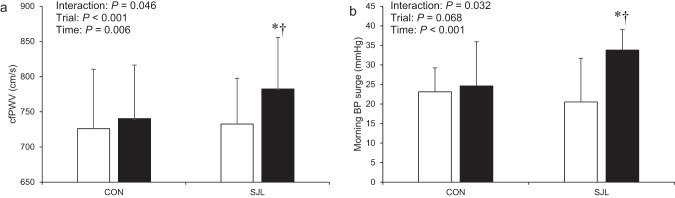
Central arterial stiffness (**a**), morning blood pressure surge (**b**) on Friday and Monday morning in both trials (n = 14). White bar: Friday. Black bar: Monday. BP, blood pressure; cfPWV, carotid-femoral pulse wave velocity; CON, control trial; SJL, social jetlag trial. Values are presented as mean ± standard deviation. ^*^Significantly different from Friday in the SJL trial (*P* < 0.05). ^†^Significantly different from Monday in the CON trial (*P* < 0.05)

Figure 2 summarizes the data on cfPWV and morning BP surge on Friday and Monday morning in both trials. There were significant trial × time interactions in these variables (cfPWV: *F* = 4.874, *P* = 0.046, Fig. 2a; morning BP surge: *F* = 5.796, *P* = 0.032, Fig. 2b). Significant differences in both variables were also observed between Monday in the SJL trial (cfPWV: *P* = 0.001; morning BP surge: *P* < 0.001) and Monday in the CON trial (cfPWV: *P* = 0.007; morning BP surge: *P* = 0.007). However, there was no significant trial × time interaction in the cfPWV and morning BP surge (cfPWV: *F* = 0.718, *P* = 0.409, n = 20; morning BP surge: *F* = 3.193, *P* = 0.092, n = 18) when including those who did not adhere to the intervention in the SJL trial.

### Association between changes in central arterial stiffness, morning BP surge and sympathetic nerve activity in vessels

**Fig. 3:**
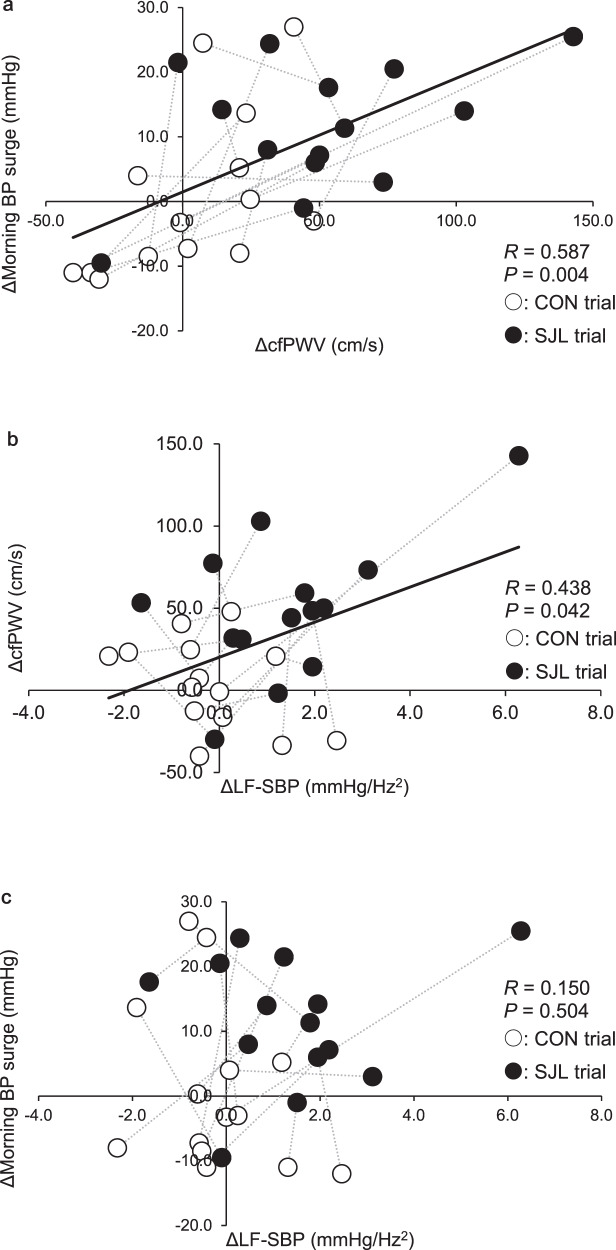
Association between changes in central arterial stiffness and morning blood pressure surge (**a**) and changes in sympathetic nerve activity and central arterial stiffness (**b**) or morning blood pressure surge (**c**) from Friday to Monday (CON trial: n = 14, SJL trial: n = 14). Δ means the change from Friday to Monday in each trial. Open circle (○): CON trial. Closed circle (●): SJL trial. The open and closed circles of each participants were connected with thin dot line. BP, blood pressure; cfPWV, carotid-femoral pulse wave velocity; CON, control trial; LF-SBP, low-frequency systolic blood pressure; SJL, social jetlag trial

Figure 3 shows an association between ΔcfPWV and Δmorning BP surge (Fig. 3a) or ΔLF-SBP (Fig. 3b) and Δmorning BP surge and ΔLF-SBP (Fig. 3c). A significant positive correlation was found between ΔcfPWV and Δmorning BP surge (*R* = 0.587, *P* = 0.004, Fig. 3a) and ΔLF-SBP (*R* = 0.438, *P* = 0.042, Fig. 3b). However, no significant correlation was found between Δmorning BP surge and ΔLF-SBP (*R* = 0.150, *P* = 0.504, Fig. 3c). These relationships were nearly unchanged when including those who did not adhere to the intervention in the SJL trial (ΔcfPWV and Δmorning BP surge: *R* = 0.605, *P* < 0.001, n = 18; ΔcfPWV and ΔLF-SBP: *R* = 0.375, *P* = 0.046, n = 20; Δmorning BP surge and ΔLF-SBP: *R* = 0.219, *P* = 0.200, n = 18).

## Discussion

The significant finding of the present study was that acute SJL on weekends (181 ± 24 min) increased cfPWV and morning BP surge on Monday (Figs. 1, 2a, b). Furthermore, a significant positive correlation was found between ΔcfPWV and Δmorning BP surge (Fig. 3a). These results were obtained without the changes in other lifestyle variables such as sleep duration, energy expenditure, and intake. Therefore, the present study suggests that acute SJL on weekends augments morning BP surge with central arterial stiffening on Monday.

### Alteration of morning BP surge by acute social jetlag

SJL trial significantly augmented morning BP surge (Fig. 2b), and the change was correlated with ΔcfPWV (Fig. 3a). Previous studies demonstrated that morning BP surge was significantly correlated with cfPWV [23, 33, 34]. Central arteries expand and recoil effectively within each cardiac cycle, providing a Windkessel effect to dampen left ventricular ejection pressure. Increased central arterial stiffness leads to less Windkessel function and augments BP [35]. Thus, the augmentation of morning BP surge in the SJL trial (Fig. 3b) might be attributed to the loss of Windkessel function caused by increased central arterial stiffness (Fig. 3a).

As baroreceptors, located in the carotid artery and aortic arch, are stretch receptors, the deformation of the barosensory arterial wall is required to initiate neural firing [36]. Knutsen et al. [37]. have suggested that the arterial diameter experiences less distension in a stiffer artery. Morning BP surge was negatively correlated with cvBRS, which modulates cardiac BP via the autonomic nervous system [38]. Moreover, it was found that a decreased cvBRS was caused by an augmentation in central arterial stiffness in the morning [39]. In the present study, the cvBRS was not significantly different between pre- and post-interventions in the SJL trial (Table 3), suggesting no influence of cvBRS on the augmentation of morning BP surge by acute SJL. Okada et al. [23]. have reported that the sympathetic baroreflex, which regulates BP via vasoconstriction, has greater contribution to the morning BP control than cvBRS. Thus, sympathetic BRS may play a more important role in the regulation of morning BP. However, we did not assess the sympathetic BRS in the present study.

### Response of central arterial stiffness to acute SJL

The cfPWV was significantly elevated during the SJL trial compared with pre-intervention (Fig. 2a). Central arterial stiffness is mainly determined by central arterial structure (e.g., elastin, collagen, and smooth muscle) and function (e.g., vasoactive substance and sympathetic nerve activity). LF-SBP, an accepted marker of sympathetic vasomotor control, was increased in the SJL trial (Table 3), and the change was positively associated with the alteration of central arterial stiffening after acute SJL (Fig. 3b). A previous study has reported that elevation of sympathetic nerve activity in vessels (measured muscle sympathetic nerve activity) increases central arterial stiffness [40]. Acute stimulation, such as exercise, drastically increases sympathetic nerve activity [41, 42]. Since acute SJL is unlikely to induce structural remodeling as suggested in several studies that investigated the effects of acute stimulation on arterial structure [43, 44], the functional component would be attributed to the temporal central arterial stiffening.

The mechanisms underlying acute SJL elevation of sympathetic nerve activity remain unclear. We can speculate that the phenomenon is attributed to circadian disruption caused by acute SJL. An animal study has shown that simulated 12-h jet lag caused by air travel led to a pressor response by increasing the activity of the sympathetic nervous system via oxidative stress in the rostral ventrolateral medulla, which plays a role in cardiovascular regulatory nuclei within the brainstem [8]. However, we did not measure any index to clarify these mechanisms underlying the elevation of sympathetic nerve activity caused by acute SJL. Although alteration of the LF-SBP has been considered a marker of changes in sympathetic efferent activity to the peripheral vasculature, it is not a directly measured sympathetic nervous activity, such as muscle sympathetic nerve activity.

### Response of central arterial stiffness and morning BP surge to acute SJL when including those who did not adhere to the intervention in the SJL trial

Although there were significant interactions in cfPWV, morning BP surge, and time course of ambulatory SBP, the significant interactions disappeared when including participants who did not adhere to the intervention in the SJL trial (SJL < 2 h). The results suggest that acute small SJL ( < 2 h) does not influence cardiovascular system in healthy men. As the present study could not clarify how many hours of SJL influence the cardiovascular system, further study is necessary.

### Study limitations

This study has some limitations. First, although we randomly assigned participants to two trials, the intervention was not performed in a blinded manner, which might have caused bias in the results. Second, we did not evaluate whether acute SJL altered circadian variables, such as clock gene expression levels and circadian-regulated hormones. Therefore, the present study could not clarify whether the intervention caused circadian disruption. Third, self-reported energy intake might be underestimated because the energy intake was low relative to energy expenditure throughout the intervention. Fourth, because only healthy male participants were recruited for the present study, the generalizability of our findings to other populations is limited. Future studies should be performed to solve these limitations.

### Perspectives

Our findings may contribute to clarifying pathophysiological mechanisms to prevent the Monday morning-specific increase in cardiovascular events. It has been reported that cardiovascular events and strokes often occur on Mondays [45–47]. Furthermore, these cardiovascular events are experienced mainly in the morning [48]. Although Kimura et al. [49]. have reported that increased double product affects the Monday morning-specific increase in cardiovascular events, the mechanisms remain unclear. Considering many people work on weekdays (Monday to Friday) and take a day off on weekends (Saturday and Sunday) in modern society, our findings provide new mechanistic insight that SJL is highly likely to contribute to the Monday morning-specific increase in cardiovascular events. To our knowledge, this is the first study examining the effect of acute SJL on the cardiovascular system. Future work should be performed to understand both the acute and chronic effects of SJL on the cardiovascular system and their underlying mechanisms based on the protocol of the present study.

In conclusion, acute SJL augments morning BP surge in the present study. Although this phenomenon may correspond to increase central arterial stiffness, we cannot yet identify the mechanisms that explain the central arterial stiffening with acute SJL. The underlying physiological mechanisms and clinical implications of these findings warrant further study. However, we assert this is the first study revealing the effects of acute SJL on the cardiovascular system.

### Supplementary information

Supplementary Materials
