# Supplementary material for: Acute social jetlag augments morning blood pressure surge: a randomized crossover trial
Source: Hypertens Res. 2023 Jul 14;46(9):2179–91. doi: 10.1038/s41440-023-01360-5 (PMC10477072; doi:10.1038/s41440-023-01360-5)
Supplement: Supplementary file 1 — Supplementary Materials [file 41440_2023_1360_MOESM1_ESM.docx]

**Supplemental Materials**

Table S1 Ambulatory blood pressure and heart rate

|  | CON | | SJL | | *P* value | | |
| --- | --- | --- | --- | --- | --- | --- | --- |
|  | Friday | Monday | Friday | Monday | Interaction | Trial | Time |
| Asleep SBP, mmHg | 107 (8) | 104 (7) | 103 (6) | 107 (5) | 0.204 | 0.197 | 0.391 |
| Asleep DBP, mmHg | 63 (8) | 60 (7) | 61 (8) | 60 (8) | 0.700 | 0.599 | 0.172 |
| Sleep HR, bpm | 53 (4) | 53 (7) | 53 (6) | 55 (6) | 0.446 | 0.447 | 0.190 |
| Preawakening SBP, mmHg | 112 (8) | 110 (7) | 109 (6) | 113 (8) | 0.094 | 0.962 | 0.380 |
| Preawakening DBP, mmHg | 67 (6) | 66 (8) | 64 (6) | 71 (6) * | 0.022 | 0.443 | 0.112 |
| Preawakening HR, mmHg | 56 (8) | 55 (7) | 55 (8) | 63 (8) *† | 0.001 | 0.015 | 0.006 |
| Morning SBP, mmHg | 122 (8) | 121 (9) | 117 (10) | 130 (7) *† | 0.001 | 0.110 | 0.008 |
| Morning DBP, mmHg | 73 (8) | 69 (9) | 71 (13) | 76 (10) | 0.043 | 0.312 | 0.823 |
| Morning HR, bpm | 66 (11) | 68 (10) | 69 (15) | 71 (17) | 0.950 | 0.237 | 0.219 |
| The lowest nocturnal SBP, mmHg | 97 (7) | 96 (7) | 97 (8) | 97 (4) | 0.985 | 0.946 | 0.979 |
| Morning double product, mmHg×bpm | 7922 (1460) | 8234 (1534) | 8111 (2204) | 9319 (2548) *† | 0.048 | 0.174 | 0.048 |
| Preawakening morning BP surge, mmHg | 14 (6) | 13 (10) | 13 (9) | 16 (7) | 0.517 | 0.720 | 0.598 |
| Wakening morning BP surge, mmHg | 10 (8) | 12 (9) | 9 (7) | 17 (11) * | 0.037 | 0.436 | 0.015 |

Values are presented as mean (standard deviation). CON, control trial; DBP, diastolic blood pressure; HR, heart rate; SJL, social jetlag trial; SBP, systolic blood pressure. *Significantly different from Friday in the SJL trial (*P* < 0.05). †Significantly different from Monday in the CON trial (*P* < 0.05).

Figure S1. Flow diagram of the participants in the randomized crossover trial.

|  | | **Monday** | **Tuesday** | **Wednesday** | **Thursday** | **Friday** | **Saturday** | **Sunday** |
| --- | --- | --- | --- | --- | --- | --- | --- | --- |
| **W**  **e**  **e**  **k**  **1** | | Familiarization term for Fitbit and the application | | | Screening term to monitor free living for each participant | | | |
| **Week 2** | **EM** | Screening term to monitor free living for each participant | | |  | AMBP & HR | CON or SJL | CON or SJL |
|  | **M** |  |  |  |  | Pre-intervention measurement1 | CON or SJL | CON or SJL |
|  | **N** |  |  |  | AMBP & HR | CON or SJL | CON or SJL | AMBP & HR |
| **Week 3** | **EM** | AMBP & HR | Wash-out | |  | AMBP & HR | CON or SJL | CON or SJL |
|  | **M** | Post-intervention measurement 2 |  |  |  | Pre-intervention measurement2 | CON or SJL | CON or SJL |
|  | **N** |  |  |  | AMBP & HR | CON or SJL | CON or SJL | AMBP & HR |
| **Week 4** | **EM** | AMBP & HR | Follow-up | |  | | | |
|  | **M** | Post-intervention measurement 2 |  |  |  |  |  |  |
|  | **N** |  |  |  |  |  |  |  |

Figure S2. Scheduling of the present study (Supplement). AMBP & HR, ambulatory blood pressure, and heart rate; CON, control trial; EM, early morning; M, morning; N, night; SJL, social jetlag trial.

Figure S3. Illustration for social jetlag.

Figure S4. Definition of morning, preawakening and wakening blood pressure (BP) surge, and typical systolic blood pressure (SBP) response to social jetlag (SJL). Circle (●): SBP. Triangle (▲): diastolic blood pressure.

Figure S5. The individual’s data for the time course of ambulatory of systolic blood pressure for 2 h before and after the awake time. Open circle (▲): CON trial on Monday, closed circle (●): SJL trial on Monday. SBP, systolic blood pressure.
